# Asthma Control and Its Related Trigger Factors in Primary Health Care in the Kingdom of Bahrain

**DOI:** 10.7759/cureus.95970

**Published:** 2025-11-02

**Authors:** Fatema A Al Jerdabi, Basem Alubaidi, Mahmood Alawainati, Fadhel A Arafat, Eman I Ateya, Layla I AlHaddad, Husain I Hasan

**Affiliations:** 1 Family Medicine, Primary Health Care Centers, Manama, BHR; 2 Medicine, Royal College of Surgeons in Ireland, Manama, BHR; 3 Family Medicine, Primary Healthcare Centers, Manama, BHR; 4 Urgent Care, Outpatient Clinic, Al-Nakheel Medical Center, Manama, BHR

**Keywords:** asthma control, asthma outcomes, asthma triggers, healthcare utilization, socioeconomic factors

## Abstract

Background

Bronchial asthma (BA) is a chronic lung disease characterized by airway inflammation and variable airflow obstruction. In Bahrain and across the Gulf Cooperation Council (GCC) countries, there is a scarcity of data regarding asthma control, triggers, and associated outcomes. This study sought to evaluate the level of asthma control among Bahraini adults, identify prevalent triggers, examine related socio-demographic and clinical factors, and assess the impact of poor asthma control on health outcomes.

Methods

A cross-sectional study was conducted from April to November 2024, involving asthma patients attending primary care centers in Bahrain. Asthma control was assessed using the Asthma Control Test, and data on socio-demographics, medical history, triggers, and outcomes were collected via structured questionnaires and analyzed using the Statistical Package for the Social Sciences software.

Results

The study included 340 adult asthma patients, predominantly female (n=227, 66.8%) and Bahraini (n=321, 94.4%), with a mean age of 44.3 years. Asthma control was classified as controlled (n=195, 57.4%), partially controlled (n=77, 22.6%), and uncontrolled (n=68, 20%). No significant differences in control were found by age, sex, body mass index (BMI), smoking, or comorbidities. However, monthly family income (P=0.006) and education level (P=0.002) were significant predictors of asthma control. Uncontrolled asthma was also linked to increased hospitalizations (P=0.045), emergency visits (P<0.001), salbutamol use (P<0.001), and absenteeism (P=0.014). Common triggers included dust (56.2%), colds (45.6%), and perfumes/incense (42.3%). Exposure to pets (P=0.001), winter weather (P=0.00l), and viral infections (P=0.027) significantly worsened asthma control.

Conclusion

A significant proportion of patients in Bahrain did not achieve adequate asthma control, particularly those with lower socioeconomic status and who were exposed to certain triggers like pets, viruses, and cold weather. Uncontrolled asthma increased healthcare utilization, and led to higher rates of absenteeism, highlighting the importance of enhanced education, regular monitoring, and improved healthcare accessibility

## Introduction

Bronchial asthma (BA) is a common chronic respiratory disease that is primarily managed in primary care. It involves chronic airway inflammation, bronchial hyperresponsiveness, and variable airflow obstruction, influenced by both genetic and environmental factors. Globally, asthma affects around 300 million people and causes approximately 180,000 deaths annually, primarily due to poor control [1]. In the Gulf Cooperation Council (GCC) countries, asthma prevalence varies widely (4.7%-32%) due to demographic differences and study methods [2]. However, comprehensive data on asthma morbidity and mortality-particularly in Bahrain-are limited [2].

Despite well-established international management guidelines, asthma control remains suboptimal worldwide. A multinational study reported well-controlled asthma in only 44.1% of children, 55.4% of adolescents, and 61.1% of adults across 25 countries [3]. Similar trends exist in the GCC, with about half of asthma patients in Saudi Arabia achieving control, while only 40% do so in Bahrain’s primary care settings [4-5]. The economic burden is significant, with most healthcare costs attributed to acute care, highlighting shortcomings in preventive strategies [6].

Multiple factors contribute to poor asthma control, including medication non-adherence, improper inhaler use, treatment resistance, comorbidities, ongoing exposure to triggers, and sociodemographic variables [1, 7]. Research has demonstrated that several demographic and socioeconomic factors-including female gender, being a child or elderly, higher body mass index (BMI), lower household income, living in industrial areas, exposure to workplace irritants or allergens, unemployment, and lower educational attainment associated with uncontrolled asthma [4, 8-10].

Environmental triggers, including viral respiratory infections, airborne allergens (such as dust mites, pet dander, and pollen), cold air, emotional stress, physical exertion, air pollution, and indoor irritants, are also key contributors to asthma exacerbation. In the GCC, dust storms, humidity, microbial blooms, and industrial pollution and heavy traffic further worsen air quality [11-18]. Cold exposure and respiratory infections particularly provoke exacerbations [7, 17-18].

Uncontrolled asthma leads to reduced quality of life, frequent symptoms, increased emergency visits, preventable mortality, and socioeconomic impacts such as reduced productivity and higher healthcare costs [6, 19-21].

A major challenge in managing asthma is that patients often think their asthma is well-controlled when it is not. Several studies have highlighted this mismatch, reinforcing the need for objective tools like the asthma control test (ACT) to frameworks such as the Global Initiative for Asthma (GINA) guidelines to ensure accurate evaluation and individualized treatment planning [1, 22]. The primary aim of asthma management is to achieve long-term control-avoiding flare-ups, maintaining daily activities, and enhancing quality of life. This is accomplished by promoting consistent use of maintenance treatments, ensuring proper inhaler technique, addressing comorbidities, minimizing exposure to triggers, and limiting dependence on short-acting beta agonists (SABAs) [1, 23-24]. Although some patients may suffer from difficult-to-treat or refractory asthma, the condition is generally manageable through a comprehensive, patient-focused approach [25-26].

Given the limited availability of region-specific data-especially in Bahrain, this study aimed to evaluate asthma control levels among adult patients in primary care. It also sought to identify common environmental and clinical triggers and assess how individual characteristics relate to asthma control and its broader health implications

## Materials and methods

Study design, setting, and population

This cross-sectional study was conducted in Bahrain between April and November 2024. It included adult patients aged 18 years and above with a physician-confirmed diagnosis of BA who were currently receiving prescribed asthma medications, such as inhaled bronchodilators or corticosteroids. Exclusion criteria included individuals under 18 years of age, pregnant women, those with cognitive impairments or language barriers, as well as patients diagnosed with chronic obstructive pulmonary disease (COPD), restrictive lung diseases, or heart failure.

This study aimed to provide a descriptive analysis of asthma control and associated trigger factors within a representative sample of the Bahraini population. A formal meta-analysis or quantitative synthesis was not performed, as this was not within the scope of the study design. The primary focus was to assess prevalence and associations rather than establish causality or pooled effect estimates. Future studies may consider a formal meta-analysis to statistically summarize outcomes across broader datasets, in which case Preferred Reporting Items for Systematic Reviews and Meta-Analyses (PRISMA) guidelines and statistical peer review would be warranted.

Sample size

The required sample size was calculated based on a 95% confidence level, a 5% margin of error, and an assumed asthma prevalence of 10.8% in Bahrain, resulting in an estimated sample of 150 participants [27]. Ultimately, 340 patients were enrolled in the final study sample to increase the power.

Sampling method and data collection process

To ensure geographic representation and minimize selection bias, a multistage random sampling strategy was employed. Bahrain’s 27 government health centers were grouped by the country's four governorates, from which eight centers (two per governorate) were randomly selected. The Health Information Directorate provided a registry of adult asthma patients with physician-confirmed diagnoses based on ICD-10 codes and documented prescriptions for asthma medications. From a randomized pool of 1,000 eligible patients, 340 met the inclusion criteria and agreed to participate. Diagnosis confirmation was conducted via electronic medical records to ensure diagnostic accuracy and reduce misclassification.

Data were collected through structured telephone interviews conducted by four trained family physicians who were part of the research team. The Arabic-translated ACT was administered for Arabic-speaking participants, while the original English ACT was administered to non-Arabic speakers. All questionnaires were interviewer-administered to promote consistency and reduce response bias.

Responses were entered directly into Microsoft Forms, which automatically generated an Excel spreadsheet. A pilot study with 30 participants was conducted in March 2024. The final dataset was then imported into SPSS version 26 (Armonk, New York) for analysis.

Instrument used

A structured questionnaire was developed comprising three sections. The first section gathered demographic data, including age, sex, nationality, weight and height, income level, education, occupation, asthma duration, and family history. The second section utilized the ACT, a validated five-item tool assessing asthma symptoms, activity limitations, nighttime disturbances, rescue medication use, and perceived control over the past four weeks, with scores ranging from five to 25 to classify asthma as controlled (≥20), partially controlled (16-19), or uncontrolled (<16). The third section focused on asthma triggers and patient history, evaluating exposure to common irritants, smoking status, and vaccination history, comorbid conditions, past exacerbations, hospitalizations, absenteeism, current medications, and frequency of short-acting beta-agonist (SABA) use (Appendices 1-8).

Data analysis

Data analysis was performed using SPSS version 26. Descriptive statistics using frequencies, percentages, means, and standard deviations, and inferential tests, including Chi-square, Fisher’s exact, and t-tests, were used. Logistic regression analysis was done to identify predictors of asthma control, reporting odds ratios (ORs) with 95% confidence intervals. A P-value <0.05 was considered statistically significant.

Definitions

Bronchial asthma (BA) was operationally defined as a physician-diagnosed condition with episodic symptoms (wheezing, cough, chest tightness, or breathlessness) and objective confirmation through peak expiratory flow measurement or response to asthma treatment. Asthma control was assessed using ACT, a globally validated tool known for its reliability and cultural sensitivity. Asthma control was categorized as controlled (ACT≥20), partially controlled (ACT 16-19), and uncontrolled (ACT<16), based on ACT score cutoffs [22], as described in the Instrument Used section. Salbutamol overuse was defined as the use of more than one SABA canister per month.

## Results

The study included 340 participants, most of whom were female (n=227, 66.8%) and Bahraini nationals (n=321, 94.4%). The mean age was 44.3 ± 14.7 years, and the average BMI was 30.4 ± 7.3. Regarding socioeconomic status, over half of the participants reported a monthly household income between 300-700 Bahraini Dinars (BD) (n=190, 55.9%). Family history of BA was reported by 62.6% (n=213), although only 38.8% (n=132) had a personal history of childhood asthma. The average duration of asthma among participants was 18.3 ± 13.8 years. The most commonly reported comorbidities were gastroesophageal reflux disease (GERD) (44.7%), sinusitis (40.3%), allergic rhinitis (35.6%), and atopic dermatitis (20.6%). Table 1 summarizes the detailed sociodemographic and clinical characteristics of the participants.

**Table 1:** Sociodemographic and Clinical Characteristics of the Study Participants (N=340)

	Variable	n (%)
Age, mean± SD		44.3±14.7
Gender	Male	113(33.2)
	Female	227(66.8)
Nationality	Bahraini	321(94.4)
	Non-Bahraini	19(5.6)
Body Mass Index (BMI), mean± SD		30.4±7.3
The monthly income of the family	<300 Bahraini Dinar (BD)	48(14.1)
	300-700 BD	190(55.9)
	701-1000 BD	71(20.9)
	>1000 BD	31(9.1)
Education level	No formal education	23(6.8)
	Primary/Intermediate	58(17.1)
	Secondary	129(37.9)
	College/University	130(38.2)
Occupation	Unemployed	65(19.1)
	Housewife	78(22.9)
	Policeman	14(4.1)
	Retired	62(18.2)
	Professionals/Clerks/Administrative	60(17.6)
	Student	15(4.4)
	Auto-mechanic/Furniture	3(0.9)
	Other	43(12.6)
Smoking status	Non-smoker	250(73.5)
	Smoker	48(14.1)
	Ex-smoker	23(6.8)
	Passive smoker	19(5.6)
Family History of Asthma	Yes	213(62.6)
	No	127(37.4)
Presence of Childhood Asthma	Yes	132(38.8)
	No	208(61.2)
Comorbid conditions	Gastroesophageal Reflex	152(44.7)
	Sinusitis	137(40.3)
	Allergic Rhinitis	121(35.6)
	Atopic Dermatitis	70(20.6)
Duration of Bronchial Asthma in Years, Mean± SD		18.3±13.8

As shown in Table 2, nearly half of the participants (n=160, 47.6%) reported no impact of asthma on their daily activities, while 25.3% (n=85) experienced limitations some of the time and 9.2% (n=31) most of the time. Shortness of breath was absent in 43.8% (n=149), occurred 1-2 times per week in 32.6% (n=111), and three to six times per week in 10.6% (n=36). Nighttime or early morning symptoms were reported by 47.4% of participants, while 52.6% (n=179) experienced none. Rescue inhaler use was absent in 47.6% of participants (n=162), used two to three times per week by 12.6%, one to two times per day by 16.2% (n=55), and used frequently (≥3 times per day) by 7.1% (n=24). Regarding self-perception of asthma control, 39.1% (n=133) felt their asthma was completely controlled, 30.6% (n=104) reported it as well controlled, and only a small number considered their condition poorly controlled (n=21, 6.2%) or not controlled at all (n=5, 1.5%). The mean asthma control test (ACT) score was 19.65 ± 4.87, which places the group at the borderline between well-controlled and partially controlled asthma. Overall, 57.4% (n=195) of participants had controlled asthma, 22.6% were partially controlled, and 20% were classified as uncontrolled (Table 2).

**Table 2:** Findings of Asthma Control Test (N=340)

Asthma Control Test Questions		n (%)
How often has your asthma prevented you from completing your usual work, school, or home activities?	All the time	0(0)
	Most of the time	31(9.2)
	Some of the time	85(25.3)
	A little of the time	60(17.9)
	None of the time	160(47.6)
How often have you had shortness of breath?	More than once per day	19(5.6)
	Once a day	25(7.4)
	3-6 times/ week	36(10.6)
	1-2 times/week	111(32.6)
	None at all	149(43.8)
How often have your asthma symptoms woken you up at night or earlier than usual in the morning (such as wheezing, coughing, shortness of breath, chest tightness, or pain)?	4 or more nights/week	29(8.5)
	2-3 nights/ week	42(12.4)
	Once a week	34(10)
	Once/twice	56(16.5)
	None at all	179(52.6)
How often have you used your rescue inhaler or nebulizer medication (such as Salbutamol)?	≥ 3 times/ a day	24(7.1)
	1-2 times/ day	56(16.5)
	2-3 times / a week	43(12.6)
	Once weekly or less	55(16.2)
	Not at all	162(47.6)
How would you rate your asthma control?	Not controlled at all	5(1.5)
	Poorly controlled	21(6.2)
	Somewhat controlled	77(22.6)
	Well-controlled	104(30.6)
	Completely controlled	133(39.1)
Categories of asthma control	Controlled Asthma(ACT ≥20)	195 (57.4%)
	Partially controlled asthma(ACT 16–19)	77(22.6%)
	Uncontrolled Asthma (ACT< 16)	68 (20%)
Asthma control test, Mean± SD		19.65±4.87

Associations between asthma control and sociodemographic & clinical factors

As illustrated in Table 3, no statistically significant associations were found between asthma control status and age (P=0.968), sex (P=0.162), nationality (P=0.901), BMI (P=0.368), occupation (P=0.196), family history of asthma (P=0.110), childhood asthma history (P=0.179), smoking status (P=0.466), or asthma duration (P=0.962). However, monthly family income was significantly associated with asthma control (P=0.006), with higher income levels correlating with better control. Similarly, education level was significantly related (P=0.002), with participants holding college or university degrees exhibiting a higher prevalence of controlled asthma.

**Table 3:** Association Between Sociodemographic, Clinical Characteristics, and Asthma Control Status (N=340)

		Uncontrolled (N=68)	Partially Controlled (N=77)	Controlled (N=195)	P-value
Age, mean± SD		44.01±14.45	44.64±15.52	44.31±14.45	0.968
Gender	Male	19(16.8)	21(18.6)	73(64.6)	0.162
	Female	49(21.6)	56(24.7)	122(53.7)	
Nationality	Bahraini	64(19.9)	72(22.4)	185(57.6)	0.901
	Non-Bahraini	4(21.1)	5(26.3)	10(52.6)	
Body Mass Index (BMI), mean± SD		31.54±7.55	30±8.36	30.2±6.84	0.368
Monthly income of the family	<300 BD	14(29.2)	14(29.2)	20(41.7)	0.006
	300-700 BD	45(23.7)	41(21.6)	104(54.7)	
	701-1000 BD	7(9.9)	13(18.3)	51(71.8)	
	>1000 BD	2(6.5)	9(29)	20(64.5)	
Education level	Illiterate	5(21.7)	5(21.7)	13(56.5)	0.002
	Primary/Intermediate	18(31)	11(19)	29(50)	
	Secondary	35(27.1)	27(20.9)	67(51.9)	
	College/University	10(7.7)	34(26.2)	86(66.2)	
Occupation	Unemployed	15(23.1)	17(26.2)	33(50.8)	0.196
	Housewife	23(29.5)	17(21.8)	38(48.7)	
	Policeman	4(28.6)	2(14.3)	8(57.1)	
	Student	1(6.7)	3(20)	11(73.3)	
	Professionals/Clerks/Administrative	6(10)	14(23.3)	40(66.7)	
	Retired	8(12.9)	14(22.6)	40(64.5)	
	Auto-mechanic	1(33.3)	0(0)	2(66.7)	
	Other	10(23.3)	10(23.3)	23(53.5)	
Family history of asthma	Yes	46(21.6)	54(25.4)	113(53.1)	0.11
	No	22(17.3)	23(18.1)	82(64.6)	
Presence of childhood asthma	Yes	33(25)	29(22)	70(53)	0.179
	No	35(16.8)	48(23.1)	125(60.1)	
Smoking status	Non-smoker	43(17.2)	59(23.6)	148(59.2)	0.466
	Ex-smoker	8(34.8)	4(17.4)	11(47.8)	
	Smoker	12(25)	10(20.8)	26(54.2)	
	Passive smoker	5(26.3)	4(21.1)	10(52.6)	
Comorbid conditions	Gastroesophageal Reflux	30(19.7)	36(23.7)	86(56.6)	0.919
	Sinusitis	26(19)	31(22.6)	80(58.4)	0.922
	Allergic Rhinitis	25(20.7)	27(22.3)	69(57)	0.973
	Allergic Dermatitis	18(25.7)	17(24.3)	35(50)	0.305
Duration of asthma in years,		18.57±12.98	18.24±13.39	18.04±14.35	0.962
Mean± SD					
Have you been admitted to the hospital for asthma attacks?	Yes	18(32.1)	11(19.6)	27(48.2)	0.045
	No	50(17.6)	66(23.2)	168(59.2)	
How many asthma attacks did you experience in the last 12 months that required an emergency care visit? Only one answer needed	0	20(11.4)	39(22.3)	116(66.3)	<0.001
	1	10(13.7)	14(19.2)	49(67.1)	
	2	10(26.3)	15(39.5)	13(34.2)	
	3 or more	28(51.9)	9(16.7)	17(31.5)	
Number of Salbutamol canisters usage per month	Not using at all	15(10.1)	26(17.6)	107(72.3)	<0.001
	<1	11(10.4)	25(23.6)	70(66)	
	1	30(44.8)	21(31.3)	16(23.9)	
	≥ 2	7(50)	5(35.7)	2(14.3)	
Number of days of school or work missed in the last year due to asthma symptoms, mean ±SD		6.17±14.15	2±5.05	2.02± 3.49	0.014

There were no significant associations between asthma control and comorbid conditions such as GERD (P=0.919), sinusitis (P=0.922), allergic rhinitis (P=0.973), or atopic dermatitis (P=0.305). Conversely, previous hospitalization due to asthma (P=0.045) and emergency department visits in the past 12 months (P<0.001) were significantly more common among those with uncontrolled asthma. The number of Short-Acting Beta-2 Agonist (SABA) canisters used per month was significantly linked to poorer asthma control, with the use of more than one canister per month strongly associated with uncontrolled asthma (P<0.001). Furthermore, the number of missed school or work days over the past year due to asthma symptoms was significantly higher among uncontrolled patients (P=0.014).

Asthma triggers and their association with asthma control

Table 4 outlines the most frequently reported asthma triggers. Dust was the most common (n=191, 56.2%), followed by common cold (n=155, 45.6%) and perfumes/incense (n=129, 42.3%). Less commonly reported triggers included pets (n=41, 12.1% often), cleaning agents (n=46, 13.5% often), and mold (n=16, 4.7% often). Winter weather was reported as a frequent trigger by 31.2% of participants, and 26.3% cited physical exercise as a frequent trigger.

**Table 4:** Frequency of Exposure to Potential Asthma Triggers Among Participants (N=340)

Frequency of Exposure TN= 340	No n (%)	Rarely n (%)	Sometimes n (%)	Often n (%)
Mold*	274(80.8)	24(7.1)	25(7.4)	16(4.7)
Cleaning agents, detergents, and antiseptics	89(26.2)	89(26.2)	116(34.1)	46(13.5)
Perfumes**	47(15.4)	23(7.5)	129(42.3)	106(34.8)
Exercises*	123(36.2)	47(13.9)	80(23.6)	89(26.3)
Pets*	228(67.3)	32(9.4)	38(11.2)	41(12.1)
Dust	51(15)	62(18.2)	191(56.2)	36(10.6)
Winter weather (cold weather)	151(44.4)	21(6.2)	62(18.2)	106(31.2)
Common cold trigger	58(17.1)	30(8.8)	97(28.5)	155(45.6)

Table 5 shows that exposure to pets (P=0.001), cold weather (P=0.001), and the common cold (P=0.027) were significantly associated with poorer asthma control. Among those never exposed to pets, 64.5% had controlled asthma, compared to 37.5% (rare), 34.2% (sometimes), and 56.1% (often) in exposed groups. Similarly, 68.2% of those not exposed to cold weather had controlled asthma versus only 41.5% of those often exposed. The prevalence of controlled asthma also declined among those frequently affected by common colds-from 74.1% in those unaffected to 51.6% in those often affected. In contrast, no significant associations were found between asthma control and exposure to mold (P=0.730), cleaning agents (P=0.891), perfumes/incense (P=0.907), exercise (P=0.800), or dust (P=0.307).

**Table 5:** Association Between Frequency of Exposure to Common Triggers and Asthma Control Levels (N=340)

Frequency of Exposure to		Uncontrolled n (%)	Partially Controlled n (%)	Controlled n (%)	P value
Mold	No	52(19)	65(23.7)	157(57.3)	0.730
	Rarely	4(16.7)	5(20.8)	15(62.5)	
	Sometimes	8(32)	5(20)	12(48)	
	Often	4(25)	2(12.5)	10(62.5)	
Cleaning agents, detergents, and antiseptics	No	19(21.3)	18(20.2)	52(58.4)	0.891
	Rarely	18(20.2)	21(23.6)	50(56.2)	
	Sometimes	20(17.2)	30(25.9)	66(56.9)	
	Often	11(23.9)	8(17.4)	27(58.7)	
Incense and perfumes	No	4(8.5)	5(10.6)	38(80.9)	0.907
	Rarely	5(21.7)	3(13.0)	15(65.2)	
	Sometimes	20(15.5)	31(24.0)	78(6.5)	
	Often	32(30.2)	30(28.3)	44(41.5)	
Exercise	No	23(18.7)	27(22.0)	73(59.3)	0.800
	Rarely	12(25.5)	11(23.4)	24(51.1)	
	Sometimes	13(16.3)	21(26.3)	46(57.5)	
	Often	20(22.5)	17(19.1)	52(58.4)	
Pets	No	43(18.9)	38(16.7)	147(64.5)	0.001
	Rarely	9(28.1)	11(34.4)	12(37.5)	
	Sometimes	10(26.3)	15(39.5)	13(34.2)	
	Often	6(14.6)	12(29.3)	23(56.1)	
Dust	No	9(17.6)	10(19.6)	32(62.7)	0.307
	Rarely	11(17.7)	14(22.6)	37(59.7)	
	Sometimes	35(18.3)	46(24.1)	110(57.6)	
	Often	13(36.1)	7(19.4)	16(44.4)	
Winter weather (cold weather)	No	18(11.9)	30(19.9)	103(68.2)	0.001
	Rarely	7(33.3)	5(23.8)	9(42.9)	
	Sometimes	13(21)	10(16.1)	39(62.9)	
	Often	30(28.3)	32(30.2)	44(41.5)	
Common cold trigger	No	6(10.3)	9(15.5)	43(74.1)	0.027
	Rarely	4(13.3)	4(13.3)	22(73.3)	
	Sometimes	25(25.8)	22(22.7)	50(51.5)	
	Often	33(21.3)	42(27.1)	80(51.6)	

## Discussion

This study assessed asthma control among adult patients attending primary care centers across Bahrain. The results showed that nearly 45% of patients had uncontrolled or partly controlled asthma, with the overall mean ACT score suggesting suboptimal asthma management.

These findings align with a similar study from Saudi Arabia, where approximately half of the patients had controlled asthma [3]. Comparatively, Qatar reported a lower control rate at 41.1% [28], indicating that Bahrain may be performing slightly better, potentially due to improved access to primary care and asthma education programs.

A notable finding was the discrepancy between patients’ perceived and actual asthma control. While around 70% believed their asthma was well or completely controlled, only 57% met the ACT criteria for control. This is consistent with earlier studies showing patients often overestimate their control [15-16, 22]. This gap highlights the importance of integrating objective tools like the ACT into routine clinical practice to ensure accurate assessment and timely treatment adjustments.

Sociodemographic factors such as higher income and university-level education were significantly associated with better asthma control. This supports global evidence that socioeconomic resources improve healthcare access, medication adherence, and health literacy, contributing to better outcomes [8-10]. Other variables-age, sex, BMI, smoking status, and asthma history-did not show significant associations in this study, possibly due to sample characteristics and lower smoking prevalence (14.1%).

Exposure to environmental factors like cold weather and viral infections was linked to uncontrolled asthma. These findings corroborate previous studies demonstrating that cold air can induce bronchoconstriction [17] and that viral respiratory infections are among the most frequent asthma exacerbation triggers [18-19]. Pet exposure was also significantly associated with poor asthma control, echoing findings from the United States Environmental Protection Agency [13] and other research identifying pet dander as a common asthma trigger. Conversely, although many participants reported mold, cleaning agents, perfumes/incense, and dust as triggers, no statistically significant associations with asthma control were observed. These results differ from prior studies linking indoor air pollutants to asthma exacerbations [14, 16], possibly due to differences in exposure levels, environmental contexts, or self-reporting bias.

Physical activity showed no significant association with asthma control, suggesting that well-managed asthma patients can safely engage in exercise, consistent with prior research [15].

The study found a high prevalence of SABA use, with over half of the participants using them occasionally or frequently. SABA overuse was significantly associated with poor asthma control, consistent with findings from international studies [22-23, 29]. These findings highlight the critical need for comprehensive patient education on proper inhaler use, the role of controller medications, and regular clinical follow-up to optimize asthma management.

Uncontrolled asthma had a substantial impact on participants’ daily lives, as one third of participants reported some or frequent limitations in physical activity (n=80, 23.6%) experienced shortness of breath more than twice per week, and nearly half suffered nocturnal or early morning symptoms. These results are in line with earlier research demonstrating that poorly controlled asthma impairs quality of life and daily functioning [19].

The socioeconomic burden was also evident. Participants with poor asthma control missed an average of 6.17 days of work or school annually, compared to approximately two days among those with controlled asthma. This reflects previously documented patterns of asthma-related absenteeism and productivity loss [20]. Moreover, individuals with uncontrolled asthma experienced more frequent hospitalizations and reported three or more emergency department visits in the past year, underscoring the significant strain on acute care services. These findings are consistent with prior studies highlighting the healthcare system burden imposed by poorly controlled asthma [1, 30].

This study’s strengths include the use of the validated ACT, a sizable and representative sample, and a comprehensive analysis of asthma triggers and environmental factors. However, several limitations must be acknowledged. Most notably, the cross-sectional design precludes any causal inferences; while associations were observed, it cannot be determined whether certain factors (e.g., income, SABA overuse) directly contribute to poor control. Additionally, reliance on self-reported data introduces potential recall and reporting bias. The absence of objective clinical measures such as spirometry, biomarker data, or prescription records may limit the accuracy of asthma control classification. Finally, the lack of longitudinal follow-up constrains insights into how asthma control may change over time or respond to interventions.

Despite these limitations, the findings align with international literature and underscore areas for improvement. Linking these observations to evidence-based guidelines such as the Global Initiative for Asthma (GINA) can strengthen interpretation. For example, the observed SABA overuse directly reflects GINA’s warning against SABA-only treatment strategies and supports their recommendation for anti-inflammatory reliever therapy and controller-based regimens. Similarly, the high prevalence of perceived control despite suboptimal ACT scores aligns with GINA's emphasis on objective monitoring tools and structured follow-up. These points reinforce the urgent need to translate global recommendations into local clinical practice in Bahrain

To place asthma control within a broader behavioral health framework, the study’s findings align with chronic disease self-management theories, such as the Chronic Care Model. These frameworks highlight the importance of patient education, self-monitoring, and shared care in improving disease outcomes. Future research and intervention strategies in Bahrain may benefit from integrating such models to enhance the effectiveness of asthma management.

## Conclusions

This study revealed that a significant proportion of asthma patients in Bahrain have partially or uncontrolled asthma, particularly those with lower socioeconomic and educational levels. Poor control was associated with increased emergency room visits, hospitalizations, absenteeism, and salbutamol overuse.

While these findings cannot establish causality, they highlight critical gaps in asthma management. Consistent with GINA recommendations, there is a pressing need to strengthen patient education, ensure adherence to controller therapies, and integrate objective tools like the ACT into routine care. Additionally, addressing common but modifiable triggers (e.g., viral infections, pet exposure) through individualized action plans and environmental control strategies can further improve outcomes.

Future research should incorporate longitudinal designs, objective clinical data, and intervention-based approaches to more accurately assess cause-and-effect relationships and evaluate the effectiveness of targeted asthma management strategies in the region.

## Figures and Tables

**Figure 1:**
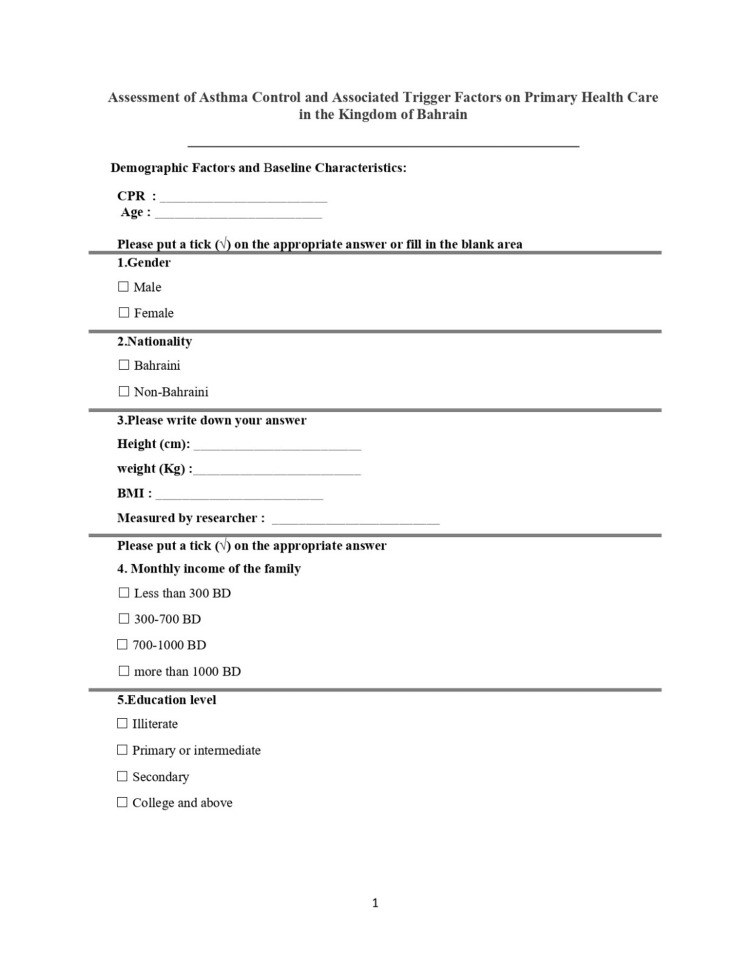
Research Questionnaire – Assessment of Asthma Control and Associated Trigger Factors at Primary Health Care in the Kingdom of Bahrain (Part 1)

**Figure 2:**
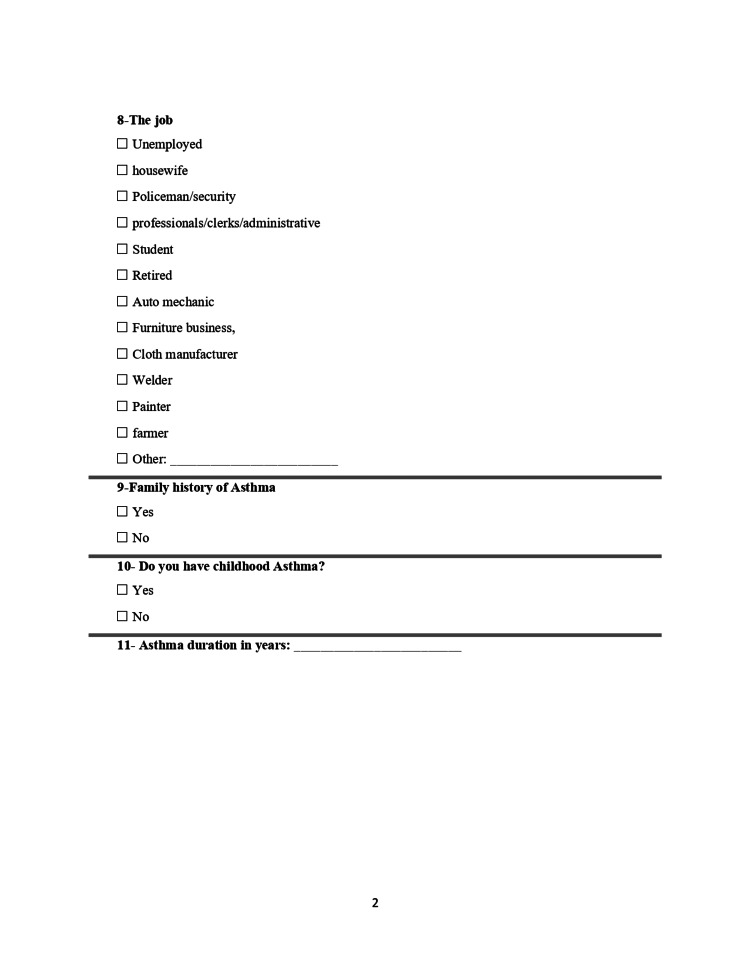
Research Questionnaire – Assessment of Asthma Control and Associated Trigger Factors at Primary Health Care in the Kingdom of Bahrain (Part 2)

**Figure 3:**
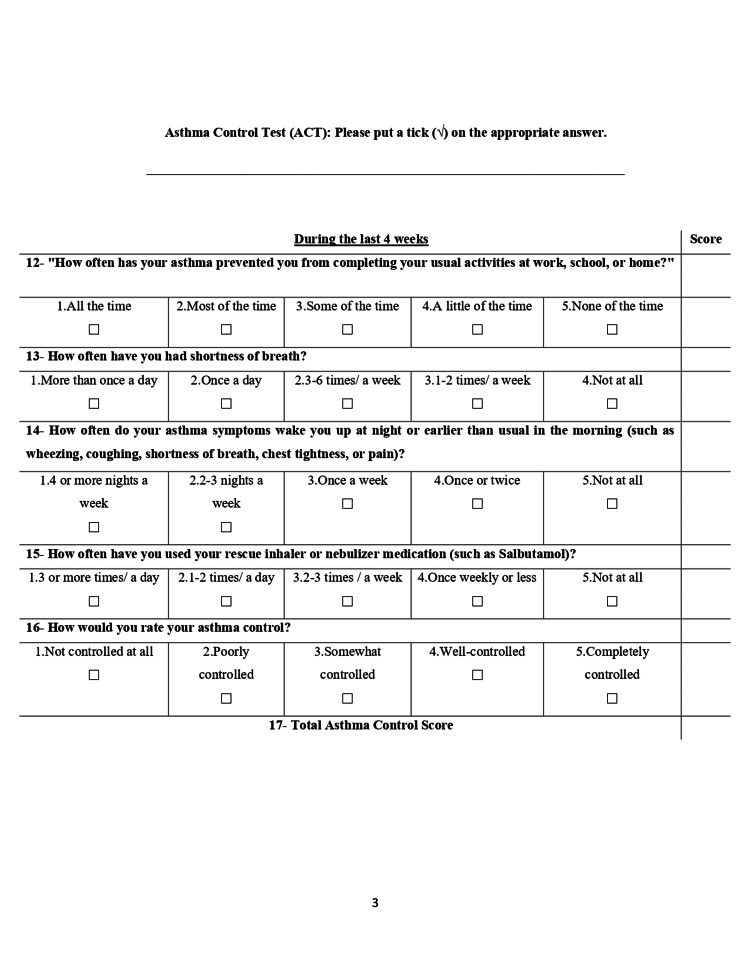
Research Questionnaire – Assessment of Asthma Control and Associated Trigger Factors at Primary Health Care in the Kingdom of Bahrain (Part 3)

**Figure 4:**
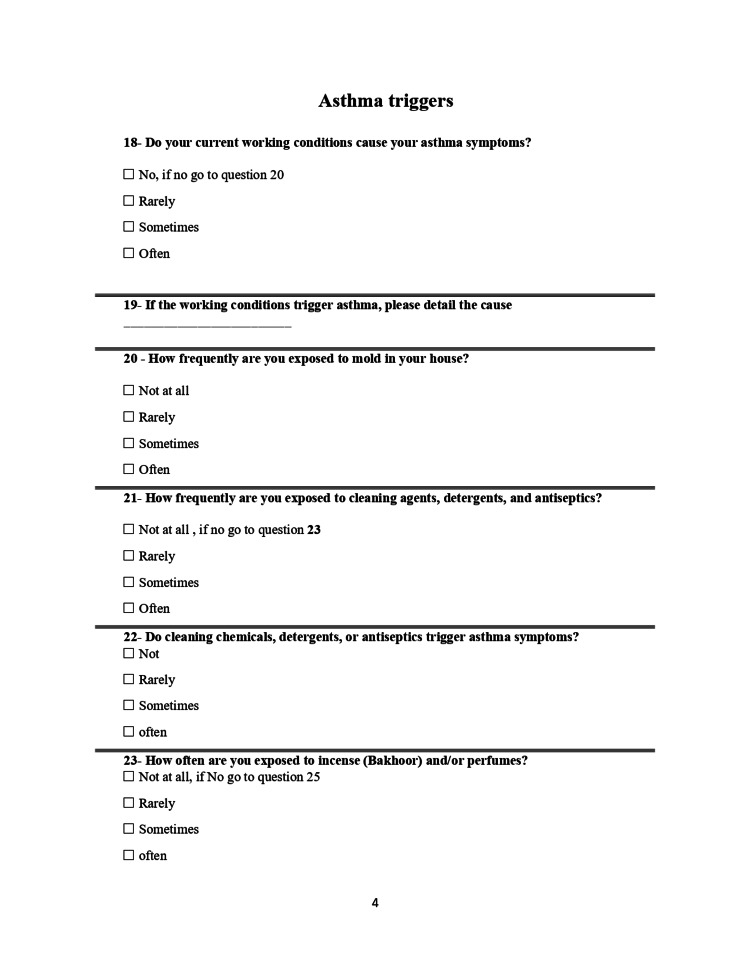
Research Questionnaire – Assessment of Asthma Control and Associated Trigger Factors at Primary Health Care in the Kingdom of Bahrain (Part 4)

**Figure 5:**
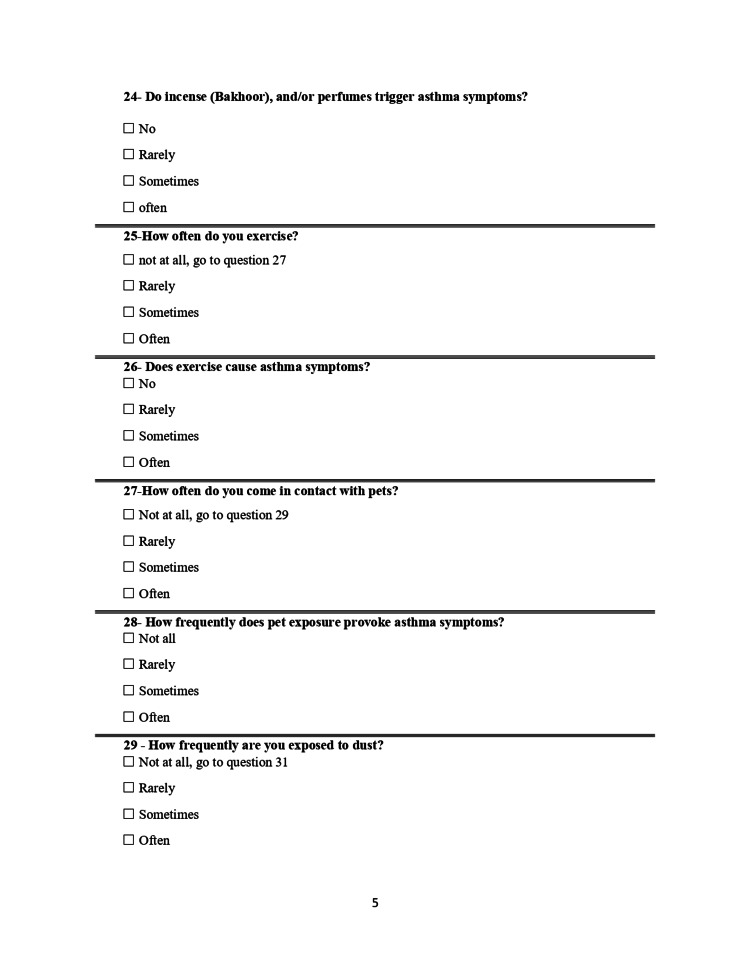
Research Questionnaire – Assessment of Asthma Control and Associated Trigger Factors at Primary Health Care in the Kingdom of Bahrain (Part 5)

**Figure 6:**
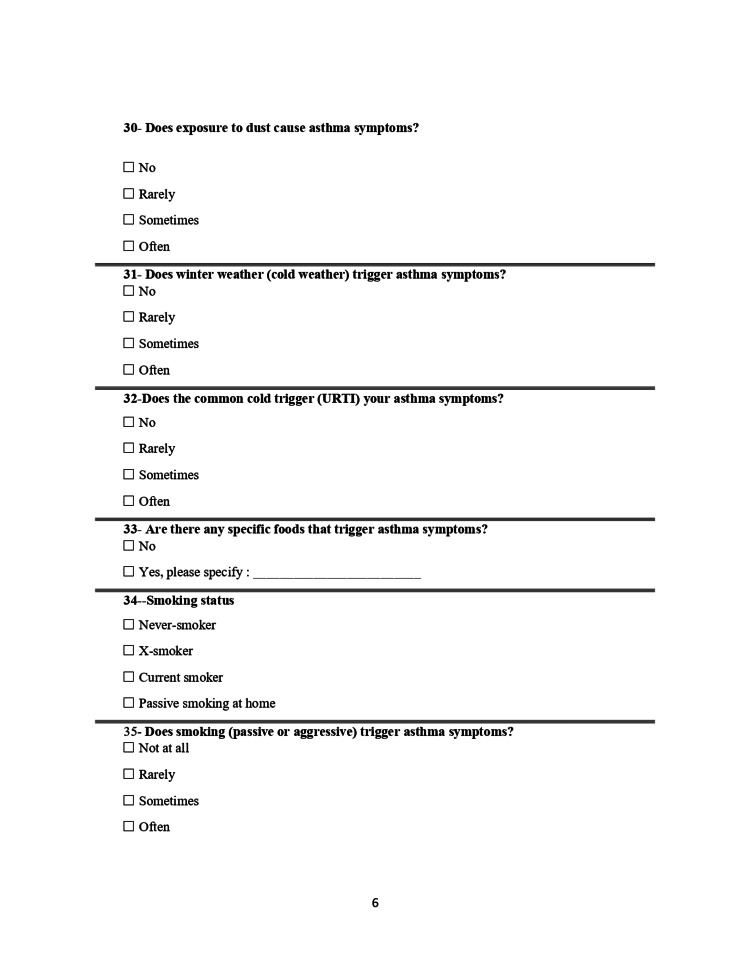
Research Questionnaire – Assessment of Asthma Control and Associated Trigger Factors at Primary Health Care in the Kingdom of Bahrain (Part 6)

**Figure 7:**
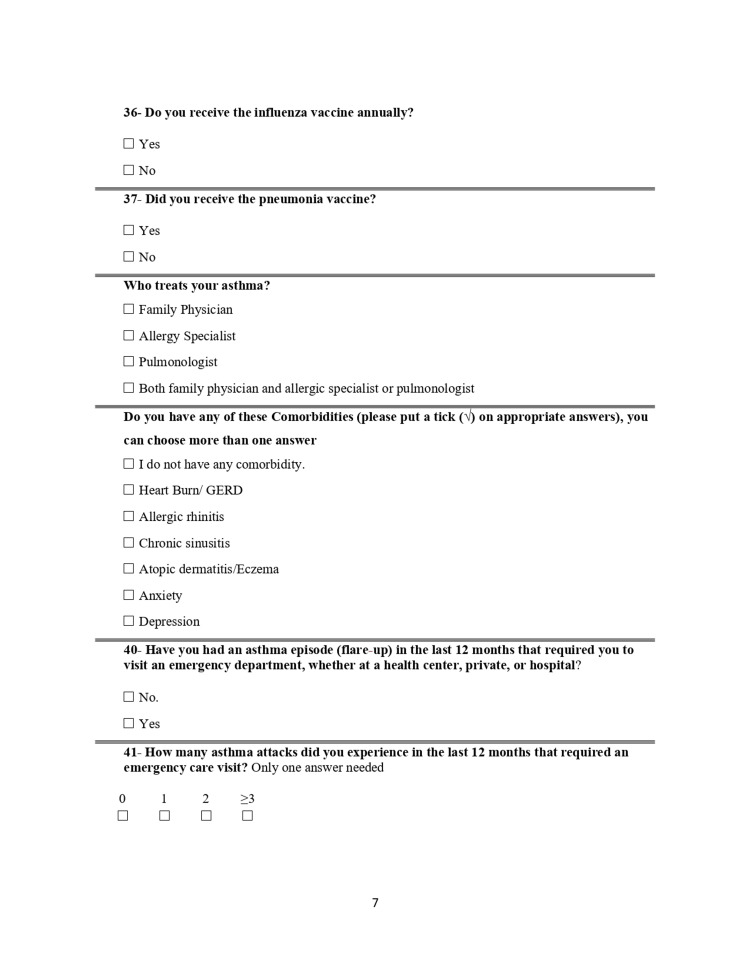
Research Questionnaire – Assessment of Asthma Control and Associated Trigger Factors at Primary Health Care in the Kingdom of Bahrain (Part 7)

**Figure 8:**
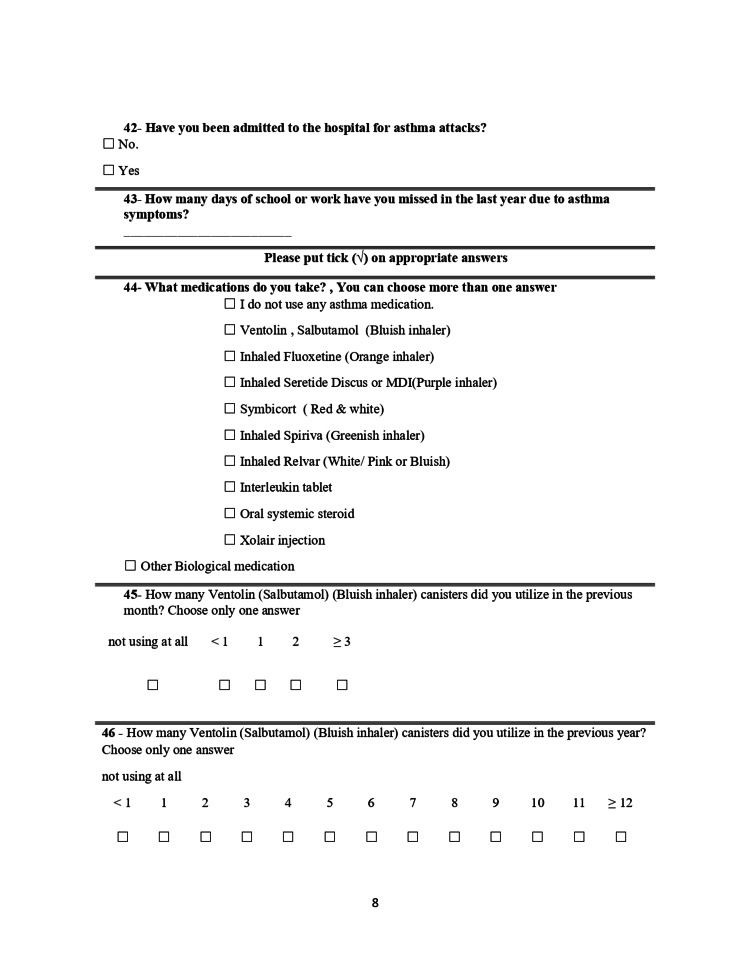
Research Questionnaire – Assessment of Asthma Control and Associated Trigger Factors at Primary Health Care in the Kingdom of Bahrain (Part 8)

## Appendices

Appendix 1

Appendix 2 

Appendix 3 

Appendix 4 

Appendix 5 

Appendix 6 

Appendix 7 

Appendix 8 
